# Molecular source attribution

**DOI:** 10.1371/journal.pcbi.1010649

**Published:** 2022-11-17

**Authors:** Elisa Chao, Connor Chato, Reid Vender, Abayomi S. Olabode, Roux-Cil Ferreira, Art F. Y. Poon

**Affiliations:** 1 Department of Pathology and Laboratory Medicine, Western University, London, Ontario, Canada; 2 School of Medicine, Queen’s University, Kingston, Ontario, Canada; University of Virginia, UNITED STATES

This is a “Topic Page” article for *PLOS Computational Biology*.

In the field of epidemiology, **source attribution** refers to a category of methods with the objective of reconstructing the transmission of an infectious disease from a specific source, such as a population, individual, or location. For example, source attribution methods may be used to trace the origin of a new pathogen that recently crossed from another host species into humans, or from one geographic region to another. It may be used to determine the common source of an outbreak of a foodborne infectious disease, such as a contaminated water supply. Finally, source attribution may be used to estimate the probability that an infection was transmitted from one specific individual to another, *i*.*e*., "who infected whom".

Source attribution can play an important role in public health surveillance and management of infectious disease outbreaks. In practice, it tends to be a problem of statistical inference, because transmission events are seldom observed directly and may have occurred in the distant past. Thus, there is an unavoidable level of uncertainty when reconstructing transmission events from residual evidence, such as the spatial distribution of the disease. As a result, source attribution models often employ Bayesian methods that can accommodate substantial uncertainty in model parameters.

Molecular source attribution is a subfield of source attribution that uses the molecular characteristics of the pathogen — most often its nucleic acid genome — to reconstruct transmission events. Many infectious diseases are routinely detected or characterized through genetic sequencing, which can be faster than culturing isolates in a reference laboratory and can identify specific strains of the pathogen at substantially higher precision than laboratory assays, such as antibody-based assays or drug susceptibility tests. On the other hand, analyzing the genetic (or whole genome) sequence data requires specialized computational methods to fit models of transmission. Consequently, molecular source attribution is a highly interdisciplinary area of molecular epidemiology that incorporates concepts and skills from mathematical statistics and modeling, microbiology, public health and computational biology.

There are generally two ways that molecular data are used for source attribution. First, infections can be categorized into different "subtypes" that each corresponds to a unique molecular variety, or a cluster of similar varieties. Source attribution can then be inferred from the similarity of subtypes. Individual infections that belong to the same subtype are more likely to be related epidemiologically, including direct source-recipient transmission, because they have not substantially evolved away from their common ancestor. Similarly, we assume the true source population will have frequencies of subtypes that are more similar to the recipient population, relative to other potential sources. Second, molecular (genetic) sequences from different infections can be directly compared to reconstruct a phylogenetic tree, which represents how they are related by common ancestors. The resulting phylogeny can approximate the transmission history, and a variety of methods have been developed to adjust for confounding factors.

Due to the associated stigma and the criminalization of transmission for specific infectious diseases, molecular source attribution at the level of individuals can be a controversial use of data that was originally collected in a healthcare setting, with potentially severe legal consequences for individuals who become identified as putative sources. In these contexts, the development and application of molecular source attribution techniques may involve trade-offs between public health responsibilities and individual rights to data privacy.

## Microbial subtyping

**Fig 1:**
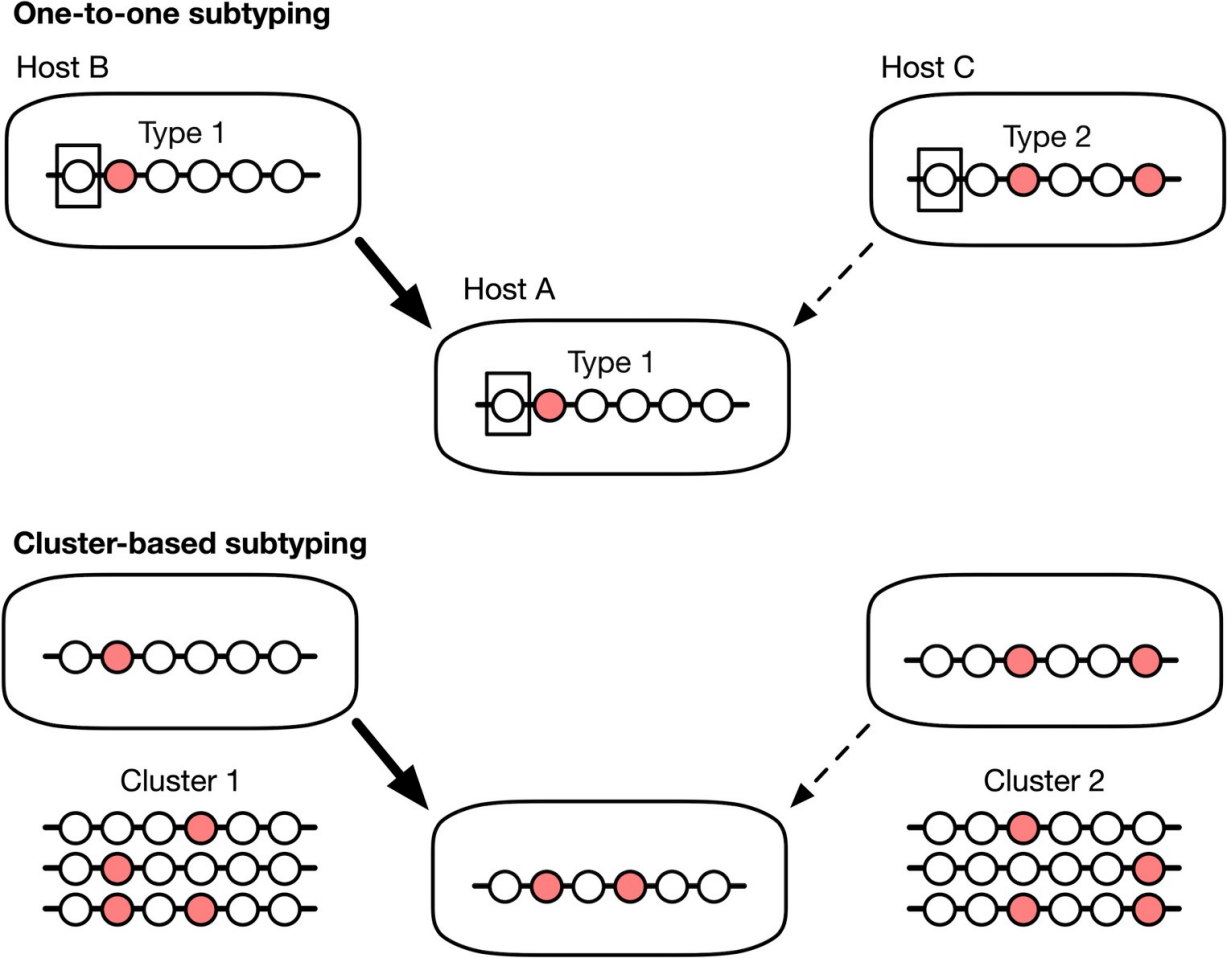
One-to-one versus cluster-based subtyping. Each set of circles along a line segment represents a molecular sequence from an individual infection. Circles are coloured to differentiate sequences. (top) Under one-to-one subtyping, every unique sequence represents a different subtype. This strategy is more common single-locus typing or pathogens with a relatively slow rate of evolution, which limits the variation that accumulates over time. (bottom) With cluster-based subtyping, multiple sequences are assigned to the same subtype. This strategy is favoured under multi-locus or whole-genome sequencing or rapidly evolving pathogens, where every sampled infection may have a unique sequence.

Microbial subtyping or strain typing is the use of laboratory methods to assign microbial samples to subtypes, which are predefined classifications based on distinct characteristics [1]. The assignment of specimens to subtypes can provide a basis of source attribution, since we assume that a pathogen undergoes minimal change when transmitted to an uninfected host. Therefore, infections of the same subtype are implied to be epidemiologically related, *i*.*e*., linked by one or more recent transmission events. The assumption that the pathogen is unchanged when transmitted is generally reasonable if the rate of evolution for the pathogen is slower than the rate of transmission, such that few mutations are observed on an epidemiological time scale [2]. For example, suppose host A is infected by a pathogen that we have categorized as subtype 1. They are more likely to have been infected by host B, who also carries the subtype 1 pathogen, than host C who carries the subtype 2 pathogen (**Fig 1**). In other words, transmission from host B is a more parsimonious explanation if there is a relatively small probability that the pathogen population in host C evolved from subtype 1 to subtype 2 after transmission to host A.

Today it is more common to use genetic sequencing to characterize the microbial sample at the level of its nucleotide sequence by sequencing the whole genome or proportions thereof [3]. However, other molecular methods such as restriction length fragment polymorphism [1] have historically played an important role in microbial subtyping before genetic sequencing became an affordable and ubiquitous technology in reference laboratories. Sequence-based typing methods confer an advantage over other laboratory methods (such as serotyping or pulsed-field gel electrophoresis [4]) because there is an enormous number of potential subtypes that can be resolved at the level of the genetic sequence. Consider the above example again; however, this time host A carries the same infection subtype as many other hosts. In this case we would have no information to differentiate between these hosts as the potential source of host A’s infection. Our ability to identify potential sources, therefore, depends on having a sufficient number of different subtypes. However, defining too many subtypes in the population makes it likely that every individual carries a unique subtype, especially for rapidly-evolving pathogens that can accumulate high levels of genetic diversity in a relatively short period of time. Hence, there exists an intermediate level of subtype resolution that confers the greatest amount of information for source attribution [5]. When source attribution is considered for a pathogen with high diversity, such that most specimens have unique genetic sequences, it is useful to group multiple unique sequences with a clustering method.

## Single and multi-locus typing

Before whole-genome sequencing was cost-effective, targeting a specific part of the pathogen genome (a.k.a. single-locus typing) was an important step to facilitate microbial subtyping. For example, the ribosomal gene 16S is a standard target for identifying bacteria, in part because it is present across all known species and contains a mixture of conserved and variable regions [6]. Within a pathogen species, sequencing targets tended to be selected on the basis of their length, ubiquity and exposure to diversifying selection, which may be dictated by the function of the gene product for expressed regions. For example, so-called "housekeeping" or core genes have indispensable biological functions, such as copying genetic material or building proteins. These genes are often preferred candidates for microbial subtyping because they are less likely to be absent from a given genome [7]. Gene presence/absence is particularly relevant for bacteria where genetic material is frequently exchanged through horizontal gene transfer.

Targeting multiple regions (loci) of the pathogen genome confers greater precision to distinguish between lineages, since the chance to observe informative genetic differences between infections is increased. This approach is referred to as multi-locus sequence typing (MLST) [8]. Similar to single-locus typing, MLST requires the selection of specific loci to target for sequencing. Moreover, for subtyping to be consistent across laboratories a reference database must be maintained that maps sequences from single or multiple loci to a fixed notation of allele numbers or designations [9].

## Whole genome sequencing

Although single- and multiple-locus subtyping is still predominantly used for molecular epidemiology, ongoing improvements in sequencing technologies and computing power continue to lower the barrier to whole-genome sequencing. Next-generation sequencing  (NGS) technologies provide cost-effective methods to generate whole genome sequences from a given sample by individually amplifying and sequencing templates in parallel using customized technologies such as sequencing-by-synthesis [10]. Shotgun sequencing applications of NGS generate full-length genome sequences by shearing the nucleic acid extracted from the sample into small fragments that are converted into a sequencing library, and then using a *de novo* sequence assembler program the genome sequence is reconstituted from the sequence fragments (short reads) [11]. Alternatively, short reads can be mapped to a reference genome sequence that has been converted into an index for efficient lookup of exact substring matches. This approach can be faster than *de novo* assembly, but relies on having a reference genome that is sufficiently similar to the genome sequence of the sample. While NGS makes it feasible to simultaneously generate full-length genome sequences from hundreds of pathogen samples in a single run, it introduces a number of other challenges. For instance, NGS platforms tend to have higher sequencing error rates than conventional sequencing, and regions of the genome with long stretches of repetitive sequence can be difficult to reassemble.

Whole genome sequencing (WGS) can confer a significant advantage for source attribution over single- or multiple-locus subtyping. Sequencing the entire genome is the maximal extent of multi-locus typing, in that all possible loci are covered. Having whole genome sequences will tend to make one-to-one subtyping (**Fig 1**) less useful, since most genomes will be unique by at least one mutation for rapidly evolving pathogens. Consequently, applications of WGS for source attribution at a population level will likely have to cluster similar genomes together [12].

The breadth of coverage offered by WGS is more advantageous for the epidemiology of bacterial pathogens than viruses. Bacterial genomes tend to be longer, ranging from about 10^6^ to 10^7^ base pairs, whereas virus genomes seldom exceed 10^6^ base pairs. In addition, bacteria tend to evolve at a slower rate than viruses, so mutations tend to be distributed more sparsely throughout a bacterial genome. For example, WGS data revealed differences between isolates of *Burkholderia pseudomallei* from Australia and Cambodia that had otherwise appeared to be identical by multi-locus subtyping due to convergent evolution [13]. WGS has also been utilized in several recent studies to resolve transmission networks of *Mycobacterium tuberculosis* in greater detail, because isolates with identical multi-locus subtypes (*e*.*g*., MIRU-VNTR profiles targeting 24 loci) were frequently separated by large numbers of nucleotide differences in the full genome sequence, comprising roughly 4.3 million nucleotides encoding over 4,000 genes [14,15].

## Genetic clustering

When applied to genetic sequences, a clustering method is a set of rules for assigning the sequences to a smaller number of clusters such that members of the same cluster are more genetically similar to each other than sequences in other clusters. Put another way, a clustering method defines a partition on the set of genetic sequences using some similarity measure. Clustering is inherently subjective and there are usually no formal guidelines for setting the clustering criteria. Consequently, cluster definitions can vary substantially from one study to the next. In addition, clustering is an intuitive process that can be accomplished by a wide variety of approaches; because of this flexibility, numerous different methods of genetic clustering have been described in the literature [16].

Genetic clustering provides a way of dealing with sequences from rapidly evolving pathogens, or whole genome sequences from pathogens with less divergence. In either case, there can be an enormous number of distinct genetic sequences in the data set. If each subtype must correspond to a unique sequence variant, then one could potentially have to track an unwieldy number of microbial subtypes for these pathogens when subtypes are defined on a one-to-one basis (**Fig 1**). The number of subtypes can be greatly reduced by expanding the definition of microbial subtypes from individually unique sequence variants to clusters of similar sequences [17]. For example, pairwise distance clustering is a nonparametric approach in which clusters are assembled from pairs of sequences that fall within a threshold distance of each other. The distance between sequences is computed by a genetic distance measure (a mathematical formula that maps two sequences to a non-negative real number) that quantifies the evolutionary divergence between the sequences under some model of molecular evolution.

## Frequency-based attribution

When the potential sources are populations, not individuals, then we are comparing the frequencies of subtypes in the respective populations. The most likely source population should have a subtype frequency distribution that is the most similar to the reference population. Methods that employ this approach have been referred to as "frequency-based" or "frequency-matching" models [18]. These subtypes are not necessarily derived from molecular data; for instance, these methods were originally applied to microbial strains defined by non-genetic antigenic or resistance profiling. For example, the "Dutch model" [19] was originally developed to estimate the most likely source of a number of foodborne illnesses due to *Salmonella* by comparing the relative frequencies of bacterial subtypes (based on phage typing) in different commercial livestock populations (including poultry, swine and cattle) through routine surveillance programs. For a given subtype, the expected number of human cases attributed to each source is proportional to the relative frequencies of that subtype among sources:

$$\lambda_{ij}=\frac{p_{ij}}{\sum_{j}p_{ij}}n_{i}$$

where is *p*_*ij*_ the proportion of (non-human) cases in the *j*-th source population associated with subtype *i*, and *n*_*i*_ is the number of cases of subtype *i* in the recipient (human) population. For instance, if the frequencies of subtype X among three potential sources was 0.8, 0.5 and 0.1, respectively, then the expected number of cases (out of a total of 100) from the second source is 0.5/(0.8+0.5+0.1)×100 = 35.7. This simple formula is a maximum likelihood estimator when the total force of infection from each source into the human population is uniform, *e*.*g*., the sources have equal population sizes.

**Fig 2:**
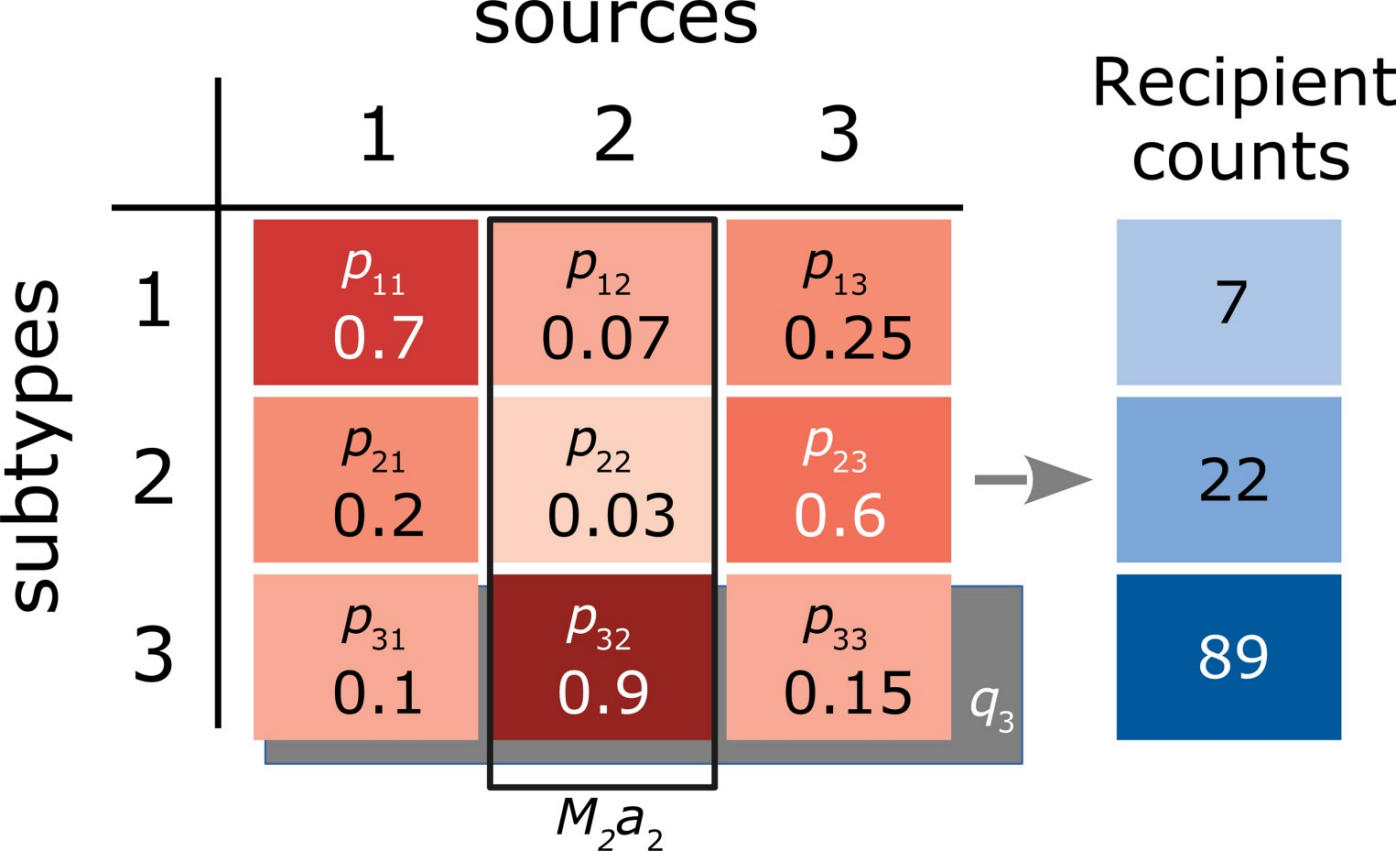
Summary of Hald model parameters. Arbitrary numbers are provided for observed quantities, such as the proportion of infections due to subtype 1 in source population 2 (*p*_12_). The marginal effect associated with source population 2 (*a*_2_) is represented by an open rectangle (solid line); while the total size of this source population *M*_2_ is observed, *a*_2_ must be estimated by regression. Similarly, the marginal effect associated with subtype 3 (*q*_3_), indicated by a rectangular shaded region) is simultaneously estimated by regression.

Subsequently, this model was extended by Hald and colleagues [20] to account for variation among sources and subtypes using Bayesian inference methods. This extension, typically referred to as the Hald model, has become a standard model in source attribution for food-borne illnesses. The observed numbers of each subtype in the human population was assumed to be a Poisson distributed outcome with a mean for the *i*-th subtype, after adjusting for cases related to travel and outbreaks:

$$\lambda_{i}=\sum_{i}\lambda_{ij}=\sum_{i}q_{i}M_{j}a_{j}p_{ij}$$

where is the marginal effect of the *i*-th subtype (*e*.*g*., elevated infectiousness of a bacterial variant), is the observed total amount (mass) of the *j*-th food source, is the marginal effect of the *j*-th food source, and is the same observed case proportion as the original "Dutch" model. This model is visualized in **Fig 2**.

## Bayesian inference

The addition of a large number of parameters to the "Dutch" model by Hald and colleagues yielded a more realistic model. However, it was too complex to solve for exact maximum likelihood estimates, in contrast to the original model. Many of the parameters could not be directly measured, such as the relative transmission risk associated with a specific food source. Consequently, Hald and colleagues adopted a Bayesian approach to estimate the model parameters. A similar approach has also been used to reconstruct the contribution of different environmental and livestock reservoirs of the bacteria *Campylobacter jejuni* to an outbreak of food poisoning in England [21], where the migration of different subtypes among reservoirs was jointly estimated by Bayesian methods.

Although Bayesian inference is discussed extensively elsewhere, it plays an important role in computationally-intensive methods of source attribution, so we provide a brief description here. In the context of Bayesian inference every parameter is described by a probability distribution that represents our *belief* about its true value. Thus, the statistical principle that underlies Bayesian inference (*i*.*e*., Bayes’ rule) can be expressed in terms of the model parameters (*θ*) and the data (*D*):

P(θ|D)∝P(D|θ)P(θ)

where P(*θ*|D), P(D|*θ*) and P(*θ*) are known as the posterior, sampling (likelihood), and prior distributions, respectively. A simple way to think about Bayesian inference is that our prior belief about the parameters is "updated" once we have seen the data. As a result, our posterior belief becomes a compromise between our prior belief and the data. To update our belief, we need to have a sampling distribution or model that describes the probability of different outcomes of an experiment. We also require a prior distribution that represents our belief in a statistical form. While modern computation allows almost any probability distribution to be used, the uniform distribution is commonly used because it assigns the same probability to every value within some range. After incorporating new information from the data, our updated belief about the model parameters is represented by the posterior distribution. This use of distributions to represent our belief distinguishes Bayesian inference from maximum likelihood, which results in a single combination of parameter values as a point estimate.

Hald and colleagues used uniform prior distributions for many of their parameters to express the prior belief that the true value fell within a continuous range with specific upper and lower limits. They constrained some parameters to take the same numerical value as others. For example, the effects of domestic and imported supplies of the same food source were linked in this manner. This assumption expressed a strong belief that a given food source carried the same transmission risk irrespective of its origin, and simplified the model so that it was more feasible to fit the data. Other parameters were set to a fixed reference value to further simplify the model.

Hald and colleagues employed a Poisson model to describe the probability of observing the number (*Y*) of rare transmission events that occur at a rate λ. As described above, the rate of cases due to a specific bacterial subtype was the sum of transmission rates across all potential sources. The Hald model was more realistic than the "Dutch" model because it allowed transmission rates to vary between subtypes and food sources. However, it was not feasible to directly measure these different rates — these parameters needed to be estimated from the data.

## Comparative methods

Instead of comparing the frequencies of subtypes to reconstruct the transmission of pathogens between populations, many source attribution methods compare the pathogen sequences at the level of individual hosts. One way of comparing sequences is to calculate some measure of genetic distance or similarity, a concept that we introduced earlier on the topic of pooling sequences into composite subtypes. For example, infections that are grouped into clusters are assumed to be related through one or more recent and rapid transmission events. Short genetic distances imply that limited time has passed for mutations to accumulate in lineages descending from their common ancestor. Consequently, these clusters are often referred to as "transmission clusters". Other studies have used genetic distances that exceed some threshold to rule out host individuals as potential sources of transmission [14,22]. Although this application of clustering is related to source attribution, it is not possible to infer the direction of transmission solely from the genetic distance between infections. Furthermore, the genetic distance separating infections is not solely determined by the rate of transmission; for example, they are strongly influenced by how infections are sampled from the population [16,23].

Sequences can also be compared in the context of their shared evolutionary history. A phylogenetic tree or phylogeny is a hypothesis about the common ancestry of species or populations. In the context of molecular epidemiology, phylogenies are used to relate infections in different hosts and are usually reconstructed from genetic sequences of each pathogen population. To reconstruct the phylogeny, the sequences must cover the same parts of the pathogen genome; for example, sequences that represent multiple copies of the same gene from different infections. It is this residual similarity (homology) between diverging populations that implies recent common ancestry. A molecular phylogeny comprises "tips" or "leaves" that represent different genetic sequences that are connected by branches to a series of common ancestors that eventually converge to a "root". The composition of the ancestral sequence at the root, the order of branching events, and the relative amount of change along each branch are all quantities that must be extrapolated from the observed sequences at the tips. There are multiple approaches to reconstruct a phylogenetic tree from genetic sequence variation [24]. For example, distance-based methods use a hierarchical clustering method to build up a tree based on the observed genetic distances.

## Phylogenetic uncertainty

**Fig 3:**
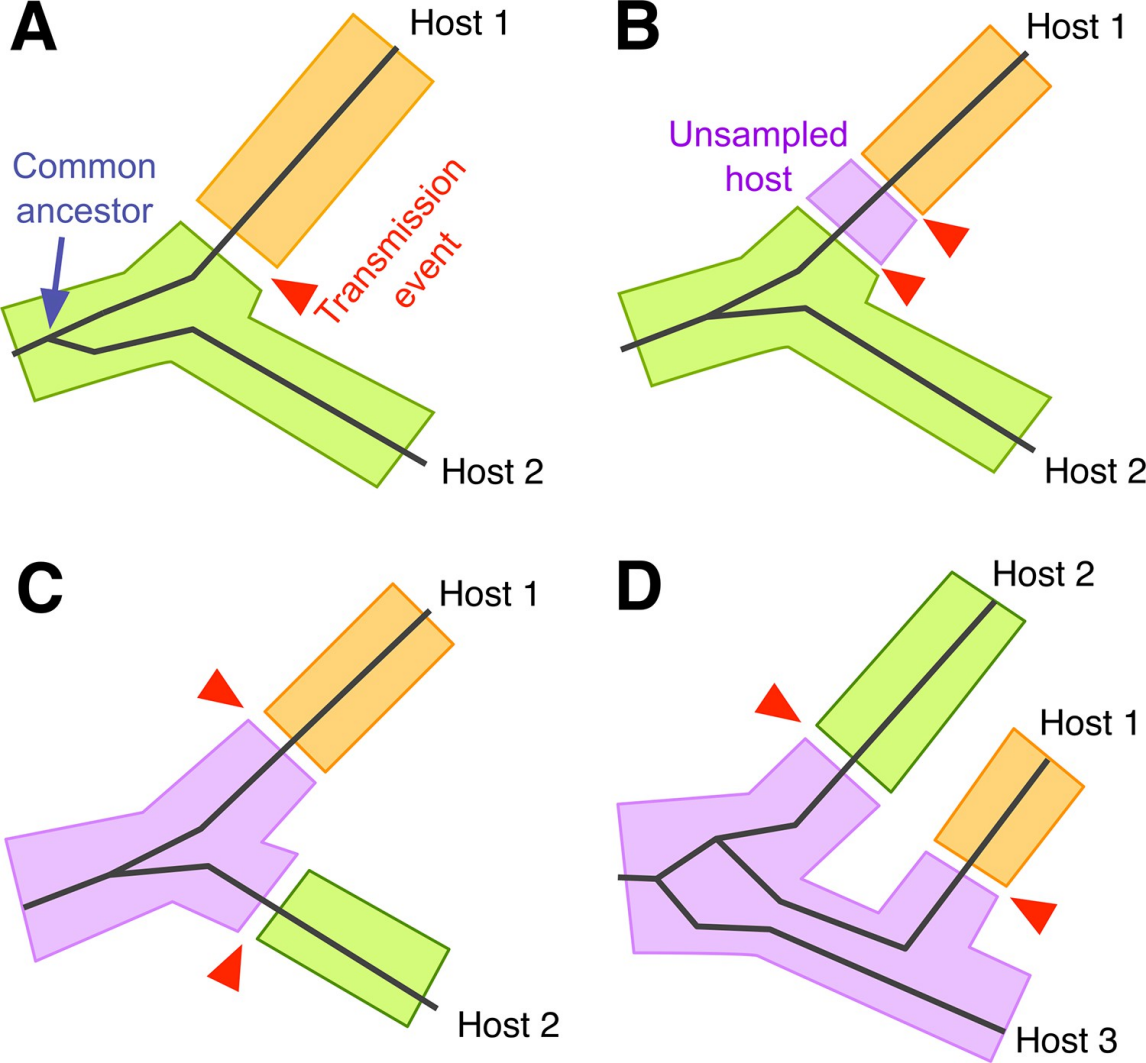
Some challenges equating phylogeny with transmission history. The solid lines represent the phylogenetic relationship between infections that have been sampled from two or three different hosts. Shaded regions correspond to the location of virus lineages in different hosts, as indicated by colour. The transmission of a lineage from one host to another is represented by a gap between shaded regions and highlighted with a red arrow. (A) Although hosts 1 and 2 are closely related, the phylogeny does not indicate whether the infection was transmitted from host 2 to 1 (as shown), or vice versa. The transmission event may be located anywhere along the two branches connecting the hosts. (B) An infection may have been transmitted through any number of unsampled hosts before reaching the host that was sampled. (C) An unsampled host may be the source of infections transmitted to both hosts 1 and 2. (D) If pathogens establish a large diverse population within each host, the branches of the phylogeny may occur in a different order than the transmission history; as shown, hosts 1 and 3 are more closely related in the transmission history, but not in the phylogeny.

A common simplifying assumption in phylogenetic investigations is that the phylogenetic tree reconstructed from the data is the "true" tree — that is, an accurate representation of the common ancestry relating the sampled infections. For instance, a single tree is often used as the input for comparative methods to detect the signature of natural selection in protein-coding sequences. On the other hand, if the phylogeny is handled as an uncertain estimate derived from the data (including the sequence alignment), then the analysis becomes a hierarchical model in which the problem of phylogenetic reconstruction is nested within the problem of estimating the other model parameters that are conditional on the phylogeny (**Fig 3**). Sampling both the phylogeny and other model parameters from their joint posterior distribution using methods such as Markov chain Monte Carlo (MCMC) should confer more accurate parameter estimates. However, the greatly expanded model space also makes it more difficult for MCMC samples to converge to the posterior distribution. Such hierarchical methods are often implemented in the software package BEAST2 [25] (Bayesian Evolutionary Analysis by Sampling Trees), which provides generic routines for MCMC sampling from tree space, and calculates the likelihood of a time-scaled phylogenetic tree given sequence data and sample collection dates.

There are a number of sources of phylogenetic uncertainty. For instance, the common ancestry of lineages can be difficult to reconstruct if there has been little to no evolution along the respective branches. This can occur when the rate of evolution is substantially slower than the time scale of transmission, such that mutations are unlikely to accumulate between the start of one infection and its transmission to the next host (*i*.*e*., the generation time). It can also arise when existing divergence is not captured due to incomplete sequencing of the respective genomes. Furthermore, reconstructing the common ancestry of lineages is progressively more uncertain as we move deeper into the tree, forcing us to extrapolate the ancestral states at greater distances from the observed data.

## Alignment

Reconstructing phylogenies from molecular sequences generally requires a multiple sequence alignment, a table in which homologous residues in different sequences occupy the same position. Although alignments are often treated as observed data known without ambiguity, the process of aligning sequences is also uncertain and can become more difficult with the rapid accumulation of sequence insertions and deletions among diverging pathogen lineages. While there are Bayesian methods that address uncertainty in alignment by joint sampling of the alignment along with the phylogeny [26], this approach is computationally complex and is seldom used in the context of source attribution. Furthermore, sequences are themselves uncertain estimates of the genetic composition of individual pathogens or infecting populations, and next-generation sequencing technologies tend to have substantially higher error rates than conventional Sanger sequencing [27], and analysis pipelines must be carefully validated to reduce the effects of sample cross-contamination and adapter contamination.

## Recombination

Genetic recombination is the exchange of genetic material between individual genomes. For pathogens, recombination can occur when a cell is infected by multiple copies of the pathogen. If some hosts were infected multiple times by two or more divergent variants from different sources (*i*.*e*., superinfection), then recombination can produce mosaic genomes that complicate the reconstruction of an accurate phylogeny [28]. In other words, different segments of a recombinant genome may be related to other genomes through discordant phylogenies in such a way that cannot be accurately represented by a single tree. In practice, it is common to screen for recombinant sequences and discard them before reconstructing a phylogeny from an alignment that is assumed to be free of recombination [29].

## Inferring transmission history from the phylogeny

The basic premise in applying phylogenetics to source attribution is that the shape of the phylogenetic tree approximates the transmission history [30], which can also be represented by a tree where each split into two branches represents the transmission of an infection from one host to another. In conjunction with reconstructing the transmission tree from other sources of information, such as contact tracing, reconstructing a phylogenetic tree can serve as a useful, additional information source especially when genetic sequences are already available. Because of the visual and conceptual similarity between phylogenetic and transmission trees, it is a common assumption that the branching points (splits) of the phylogeny represent transmission events. However, this assumption will often be inaccurate. A transmission event may have occurred at any point along the two branches that separate one sampled infection from the other in the virus phylogeny (**Fig 3A**). The transmission tree only constrains the shape of the phylogenetic tree. Thus, even if we can reconstruct the phylogenetic tree without error, there are several reasons why it will not be an accurate representation of the transmission tree, including incomplete sampling, pathogen evolution within hosts, and secondary infection of the same host.

### Incomplete sampling

Equating the phylogenetic tree with the transmission history implicitly assumes that genetic sequences have been obtained from every infected host in the epidemic. In practice, only a fraction of infected hosts is represented in the sequence data. The existence of an unknown and inevitably substantial number of unsampled infected hosts is a major challenge for source attribution. Even if the phylogenetic tree indicates that two infections are most closely related than any other sampled infection, one cannot rule out the existence of one or more unsampled hosts whom are intermediate links in the "transmission chain" separating the known hosts (**Fig 3B**). Similarly, an unsampled infection may have been the source population for both observed infections at the tips of the tree (**Fig 3C**). By itself, the phylogenetic tree does not explicitly discriminate among these alternative transmission scenarios.

### Evolution within hosts

The shape of the phylogenetic tree may diverge from the underlying transmission history because of the evolution of diverse populations of the pathogen within each host. Individual copies of the pathogen genome that are transmitted to the next host are, by definition, no longer in the source population. A split exists in the phylogenetic tree that represents the common ancestor between the transmitted lineages and the other lineages that have remained and persisted in the source population. If we follow both sets of lineages back in time, the time of the transmission event is the *most recent* possible time that they could converge to a common ancestor. Put another way, this event represents one extreme of a continuous range where the common ancestor is located further back in time.

This process is often modelled by Kingman’s coalescent [31], which describes the number of generations we expect to follow randomly selected lineages back in time until we encounter a common ancestor. The expected time until two lineages converge to a common ancestor, known as a coalescence event, is proportional to the effective population size, which determines the number of possible ancestors. Put another way, two randomly selected people in a large city are less likely to have a great-grandparent in common than two people in a small rural community.

Longer coalescence times in a large, diverse within-host pathogen populations are a significant challenge for source attribution, because it uncouples the virus phylogeny from the transmission tree. For example, if a host has transmitted their infection to two others, then there can be as many as three sets of lineages whose ancestry can be traced in the source population in that host (**Fig 3D**). As a result, there is some chance that the branching order in the virus phylogeny implies a different order of transmission events if we interpret the phylogeny as equivalent to a transmission tree. For example, in **Fig 3D** hosts 1 and 3 are more closely related in the transmission history, but not in the phylogeny.

### Clearance and secondary infection

Many infections can be spontaneously cleared by the host’s immune system. If a host that has cleared a previously diagnosed infection becomes re-infected from another source, then it is possible for the same host to be represented by different infections in the phylogenetic and transmission trees, respectively. In addition, some individuals may become infected from multiple different sources. For example, roughly one-third of infections by hepatitis C virus are spontaneously cleared within the first six months of infection [32]. This previous exposure, however, does not confer immunity to re-infection by the same virus [33]. In addition, co-infection by multiple strains of hepatitis C virus that persist simultaneously within the same host can occur relatively frequently in populations with a high rate of transmission, such as people who inject drugs using shared equipment (ranging from 14% to 39%) [34]. The persistence of strains from additional exposures may be missed by conventional genetic sequencing techniques if they are present at a low frequencies within the host, necessitating the use of "next-generation" sequencing technologies. For these reasons, the epidemiological linkage of hepatitis C virus infections through genetic similarity may be a transient phenomenon, leading some investigators to recommend using multiple virus sequences sampled from different time points of each infection for molecular epidemiology applications [29].

## Ancestral host-state reconstruction

Ancestral reconstruction is the application of a model of evolution to a phylogenetic tree to reconstruct character states, such as nucleotide sequences or phenotypes, at the different ancestral nodes of the tree down to the root [35]. In the context of source attribution, ancestral reconstruction is frequently used to estimate the geographic location of pathogen lineages as they are carried from one region to another by their hosts. Drawing this analogy between character evolution and the spatial migration of individuals or populations is known as phylogeography [36], where the geographic location of an ancestral population is reconstructed from the current locations of its sampled descendants under some model of migration.

Migration models generally fall into two categories of discrete-state and continuous-state models. Discrete-state or island migration models assume that a given lineage is in one of a finite number of locations, and that it changes location at a constant rate over time according to a continuous-time Markov process, analogous to the models used for molecular evolution. Ancestral reconstruction with a discrete-state migration model has also been utilized to reconstruct the early spread of HIV-1 in association with development of transport networks and increasing population density in central Africa [37]. Discrete models can also be applied to the population-level source attribution of zoonotic transmissions by reconstructing different host species as ancestral character states. For example, a discrete trait model of evolution was used to reconstruct the ancestral host species in a phylogeny relating *Staphylococcus aureus* specimens from humans and domesticated animals [38]. Similarly, Faria and colleagues [39] analyzed the cross-species transmission of rabies virus as a discrete diffusion process along the virus phylogeny, with rates influenced by the evolutionary relatedness and geographic range overlap of the respective host species.

Continuous-state migration models are more similar to models of Brownian motion in that a lineage may occupy any point within a defined space. Although continuous models can be more realistic than discrete migration models, they may also be more challenging to fit to data. Taken literally, a continuous model requires precise geolocation data for every infection sampled from the population. In many applications, however, these metadata are not available; for example, some studies approximate the true spatial distribution of sampled infections by the centroids of their respective regions [40]. This can become problematic if the regions vary substantially in area, and host populations are seldom uniformly distributed within regions.

## Paraphyly

Paraphyly is a term that originates from the study of cladistics, an evolutionary approach to systematics that groups organisms on the basis of their common ancestry. A group of infections is paraphyletic if the group includes the most recent common ancestor, but does not include all its descendants. In other words, one group is *nested* within an ancestral group. For example, birds are descended from a common ancestor that in turn shares a common ancestor with all reptiles; thus, birds are nested within the phylogeny of reptiles, making the latter a paraphyletic group. Thus, paraphyly is evidence of evolutionary precedence: the ancestor of all birds was a reptile. In the context of source attribution, paraphyly can be used as evidence that one infection preceded another. It does not provide evidence that the infection was directly transmitted from one individual to another, in part because of incomplete sampling.

The application of paraphyly for source attribution requires that the phylogenetic tree relates multiple copies of the pathogen from both the putative source and recipient hosts. To elaborate, phylogenetic trees relating different infections are often reconstructed from population-based sequences (direct sequencing of the PCR amplification product), where each sequence represents the consensus of the individual pathogen genomes sampled from the infected host. If copies of the pathogen genome are sequenced individually by limiting dilution protocols or next-generation sequencing, then one can reconstruct a tree that represents the genealogy of individual pathogen lineages, rather than the phylogeny of pathogen populations.

If sequences from host A form a monophyletic clade (in which members comprise the complete set of descendants from a common ancestor) that has a nested paraphyletic clade of sequences from host B, then the tree is consistent with the direction of transmission having originated from host A [41]. Directionality does not imply that host A directly transmitted their infection to host B, because the pathogen may have been transmitted through an unknown number of intermediate unsampled hosts before establishing an infection in host B.

## Node support

The statistical confidence in directionality of transmission from a given tree is usually quantified by the support value associated with the node that is ancestral to the nested monophyletic clade. The support of node *X* is the estimated probability that if we repeated the phylogenetic reconstruction on an equivalent data set, the new tree would contain exactly the same clade comprising exclusively of all descendants of node *X* in the original tree. In other words, it quantifies the reproducibility of that node given the data. It should not be interpreted as the probability that the clade below node *X* appears in the "true" tree [42]. There are generally three approaches to estimating node support:

1. *Bootstrapping*. Felsenstein adapted the concept of nonparametric bootstrapping) to the problem of phylogenetic reconstruction by maximum likelihood [43]. Bootstrapping provides a way to characterize the sampling variation associated with the data without having to collect additional, equivalent samples. To start, one generates a new data set by sampling an equivalent number of nucleotide or amino acid positions at random with replacement from the multiple sequence alignment - this new data set is referred to as a "bootstrap sample". A tree is reconstructed from the bootstrap sample using the same method as the original tree. Since we are sampling sets of homologous characters (columns) from the alignment, the information on the evolutionary history contained at that position is intact. We record the presence or absence of clades from the original tree in the new tree, and then repeat the entire process until a target number of replicate trees have been processed. The frequency at which a given clade is observed in the bootstrap sample of trees quantifies the reproducibility of that node in the original tree.

Non-parametric bootstrapping is a time-consuming process that scales linearly with the number of replicates, since every bootstrap sample is processed by the same method as the original tree, and post-processing steps are required to enumerate clades. The precision of estimating the node support values increases with the number of bootstrap replicates. For instance, it is not possible to obtain a node support of 99% if fewer than 100 bootstrap samples have been processed. Consequently, it is now more common to use faster approximate methods to estimate the support values associated with different nodes of the tree (for instance, see approximate likelihood-ratio testing below).

2. *Bayesian sampling*. Instead of using bootstrapping to resample the data, one can quantify node support by examining the uncertainty in reconstructing the phylogeny from the given data. Bayesian sampling methods such as Markov chain Monte Carlo (see Hald model) are designed to generate a random sample of parameters from the posterior distribution given the model and data. In this case, the tree is a collection of parameters. A Bayesian estimate of node support can be extracted from this sample of trees by counting the number of trees in which the monophyletic clade that descends from that specific node appears [44]. Bayesian sampling is computationally demanding because the space of all possible trees is enormous, making convergence difficult or not feasible to attain for large data sets [45].

3. *Approximate likelihood-ratio testing*. Unlike Bayesian sampling, this method is performed on a single estimate of the tree based on maximum likelihood, where the likelihood is the probability of the observed data given the tree and model of evolution. The likelihood ratio test (LRT) is a method for selecting between two models or hypotheses, where the ratio of their likelihoods is a test statistic that is mapped to a null distribution to assess statistical significance. In this application, the alternative hypothesis is that a branch in the reconstructed tree has a length of zero, which would imply that the descendant clade cannot be distinguished from its background [46]. This makes the LRT a localized analysis: it evaluates the support of a node when the rest of the tree is assumed to be true. On the other hand, this narrow scope makes the approximate LRT method computationally efficient in comparison to Bayesian sampling and bootstrap sampling. In addition to the LRT method, there are several other methods for fast approximation of bootstrap support and this remains an active area of research [47].

## Background sequences

The interpretation of monophyletic and paraphyletic clades is contingent on whether a sufficient number of infections have been sampled from the host population. Sequences from one host can only become paraphyletic relative to sequences from a second host if the tree contains additional sequences from at least one other host in the population. As noted above, there may be unsampled host individuals in a "transmission chain" connecting the putative source to the recipient host (**Fig 3B**). The incorporation of background sequences from additional hosts in the population is similar to the problem of rooting a phylogeny using an outgroup), where the root represents the earliest point in time in the tree. The location of this "root" in the section of the tree relating the sequences from the two hosts determines which host is interpreted to be the potential source.

There are no formal guidelines for selecting background sequences. Typically, one incorporates sequences that were collected in the same geographic region as the two hosts under investigation. These local sequences are sometimes augmented with additional sequences that are retrieved from public databases based on their genetic similarity (*e*.*g*., BLAST)), which were not necessarily collected from the same region. Generally, the background data comprise consensus (bulk) sequences where each host is represented by a single sequence, unlike the putative source and recipient hosts from whom multiple clonal sequences have been sampled. Because clonal sequencing is more labor-intensive, such data are usually not available to use as background sequences. The incorporation of different types of sequences (clonal and bulk) into the same phylogeny may bias the interpretation of results, because it is not possible for sequences to be nested within the consensus sequence from a single background host.

## Phylodynamic methods

In general, phylodynamics is a subdiscipline of molecular epidemiology and phylogenetics that concerns the reconstruction of epidemiological processes, such as the rapid expansion of an epidemic or the emergence of herd immunity in the host population, from the shape of the phylogenetic tree relating infections sampled from the population [48]. A phylodynamic method uses tree shape as the primary data source to parameterize models representing the biological processes that influenced the evolutionary relationships among the observed infections. This process should not be confused with fitting models of evolution (such as a nucleotide substitution model or molecular clock model) to reconstruct the shape of the tree from the observed characteristics of related populations (infections), which originates from the field of phylogenetics. The relatively rapid evolution of viruses and bacteria makes it feasible to reconstruct the recent dynamics of an epidemic from the shape of the phylogeny reconstructed from infections sampled in the present.

**Fig 4:**
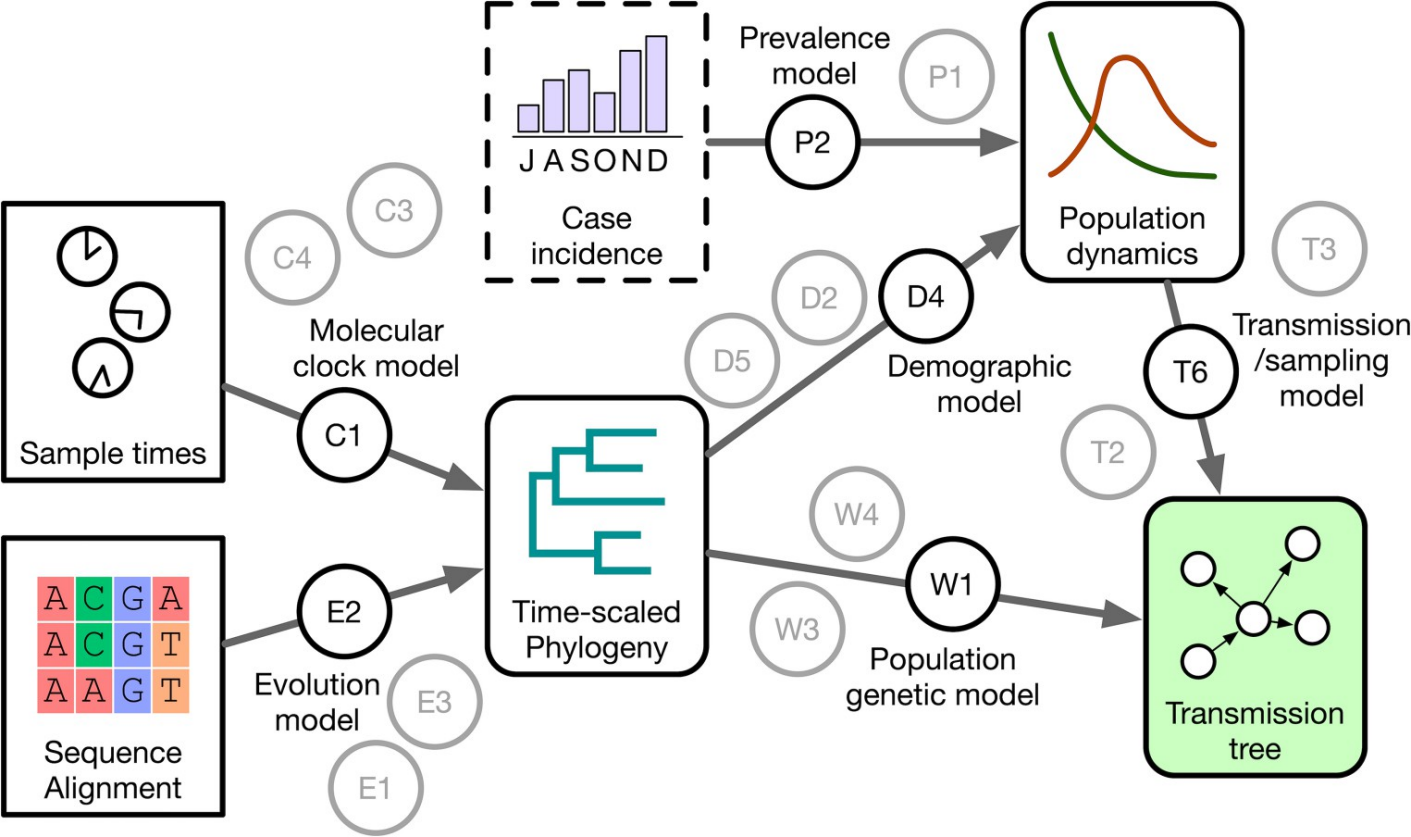
Components of a phylodynamic source attribution analysis. This diagram summarizes the structure of a phylodynamic analysis as a hierarchical Bayesian model. Rectangular nodes represent data sources (sequence alignment, sample collection times), and nodes with rounded corners represent parameter estimates (fitted models) that can in turn be used as data inputs for a subsequent model. Each arrow represents a model inference step that generates samples from the posterior distribution defined by the data and the model. The model associated with each step is represented by a circular node; each model comprises a number of prior beliefs. This schematic displays multiple model nodes to emphasize the existence of other models with different assumptions and prior beliefs that are not necessarily evaluated on the data.

The use of phylodynamic methods for source attribution involve reconstruction of the transmission tree, which cannot be directly observed, from its residual effect on the shape of the phylogenetic tree. Although there are established methods for reconstructing phylogenetic trees from the genetic divergence among pathogen populations sampled from different host individuals, there are several reasons why the phylogeny may be a poor approximation of the transmission tree (**Fig 3**). In this context, phylodynamic methods attempt to reconcile the discordance between the phylogeny and the transmission tree by modeling one or more of the processes responsible for this discordance, and fitting these models to the data (**Fig 4**).

Given the complexity of phylodynamic models, these methods predominantly use Bayesian inference to sample transmission trees from the posterior distribution, where the transmission tree is an explicit model of "who infected whom". Although these methods can estimate the probability of a direct transmission from one individual to another, this probability is conditional on how well the model (selected from a number of possible models) approximates reality. Below we describe models that have been implemented to incorporate, but not eliminate, the additional uncertainty caused by the various assumptions required when using the phylogenetic tree as an approximation of the transmission history.

## Demographic and transmission models

A basic simplifying assumption is that every infection in the epidemic is represented by at least one genetic sequence in the data set [49–51] (complete sampling). Although complete sampling may be feasible in circumstances such as an outbreak of disease transmission among farms in a defined geographic region [52], it is generally not possible to rule out unsampled sources in other contexts. This is especially true for infectious diseases that are stigmatized and/or associated with marginalized populations [53], that have a long asymptomatic period [54], or in the context of a generalized epidemic where disease prevalence may substantially exceed the local capacity for sample collection and genetic sequencing.

Several methods attempt to address the presence of unsampled hosts by modeling the growth of the epidemic over time, which predicts the total number of infected hosts at any given time. Put another way, the probability that an infection was transmitted from an unsampled source is determined in part by the total size of the infected population at the time of transmission. These models of epidemic growth are sometimes referred to as demographic models because some are derived from population growth models such as the exponential and logistic growth models. Alternatively, the number of infections can be modeled by a compartmental model that describes the rate that individual hosts switch from susceptible to infected states, and can be extended to incorporate additional states such as recovery from infection or different stages of infection [50,55]. An important distinction between population growth and compartmental models is that the number of uninfected susceptible hosts is tracked explicitly in the latter.

A phylodynamic analysis attempts to parameterize the growth model by using the phylogeny as either a direct proxy of the transmission tree, or to account for the discordance between these trees due to within-host diversity using a population genetic model, such as the coalescent (**Fig 4**). Bayesian methods make it feasible to supplement this task with other data sources, such as the reported case incidence and/or prevalence over time [56]. The transmission process can be mapped to the size of the infected population using either a coalescent (reverse-time) model or a forward-time model such as birth-death or branching processes.

Thus, the coalescent model has two different applications in phylodynamics. First, it can be used to address the confounding effect of diverse pathogen populations within hosts, by explicitly modeling the common ancestry of individual pathogens [31]. Second, the coalescent can be adapted to model the spread of infections back in time [57], drawing an analogy between the common ancestry of individuals within hosts and the transmission of infections among hosts. This parallel has also been explored by phylodynamic models based on the structured coalescent [58], where the population can be partitioned into two or more subpopulations (demes)). Each deme represents an infected host individual. Due to limited migration of pathogen lineages between demes, two pathogen lineages sampled at random are more likely to share a recent common ancestor if they belong to the same deme.

Birth-death models describe the proliferation of infections forward in time, where a "birth" event represents the transmission of an infection to an uninfected susceptible host, and a "death" event can represent either the diagnosis and treatment of an infection, or its spontaneous clearance by the host [59]. This class of models was originally formulated to describe the proliferation of species through speciation and extinction [60]. Similarly, branching processes model the growth of an epidemic forward in time where the number of transmissions from each infected host ("offspring") is described by a discrete probability distribution over non-negative integers, such as the negative binomial distribution [61]. Branching process models tend to use the simplifying assumption that this offspring distribution remains constant over time, making this class of models more appropriate for the initial stage of an epidemic where most of the population is uninfected.

## Within-host diversity

As noted above, the diversification of pathogen populations within each host results in a discordance between the shapes of the pathogen phylogeny and the transmission tree. Phylodynamic methods that treat the phylogeny as equivalent to the transmission tree assume implicitly that the population within each host is small enough to be approximated by a single lineage [48,52,62]. If the within-host population is diverse, then a transmission event will tend to underestimate the time since two lineages split from their common ancestor (**Fig 3A**); this phenomenon is analogous to the incomplete lineage sorting affecting gene trees relative to the species tree [63]. The resulting discordance between the phylogenetic and transmission trees makes it more difficult to reconstruct the latter from the observed data. Moreover, the effect of within-host diversity becomes even greater if there are incomplete transmission bottlenecks — where a new infection is established by more than one lineage transmitted from the source population — because the common ancestor of pathogen lineages may be located in previous hosts further back in time [58].

## Controversies

Source attribution is an inherently controversial application of molecular epidemiology because it identifies a specific population or individual as being responsible for the onward transmission of an infectious disease. Because molecular source attribution increasingly requires the specialized and computationally-intensive analysis of complex data, the underlying model assumptions and level of uncertainty in these analyses are often not made accessible to principal stakeholders, including the key affected populations and community advocates.

### Molecular forensics and HIV-1 transmission

Outside of a public health context, the concept of source attribution has significant legal and ethical implications for people living with HIV to potentially become prosecuted for transmitting their infection to another person. The transmission of HIV-1 without disclosing one’s infection status is a criminally prosecutable offense in many countries [64], including the United States. For example, defendants in HIV transmission cases in Canada have been charged with aggravated sexual assault, with a "maximum penalty of life imprisonment and mandatory lifetime registration as a sex offender" [65]. Molecular source attribution methods have been utilized as forensic evidence in such criminal cases.

### Forensic applications of phylogenetic clustering

One of the earliest and well-known examples of an HIV-1 transmission case was the investigation of the so-called "Florida dentist", where an HIV-positive dentist was accused of transmitting his infection to a patient. Although genetic clustering — specifically, clustering in the context of a phylogeny — was applied to these data to demonstrate that HIV-1 particles sampled from the dentist were genetically similar to those sampled from the patient [66], clustering alone is not sufficient for source attribution. Clusters can only provide evidence that infections are unlikely to be epidemiologically linked because they are too dissimilar relative to other infections in the population [67]. For example, similar phylogenetic methods were used in a subsequent case to demonstrate that the HIV-1 sequence obtained from the patient was far more similar to the sequence from their sexual partner than the sequence from a third party under investigation [68].

Clustering provides no information on the directionality of transmission (*e*.*g*., whether the infection was transmitted from individual A to individual B, or from B to A; **Fig 3**), nor can it rule out the possibility that one or more other unknown persons (from whom no virus sequences have been obtained) were involved in the transmission history. Despite these known limitations of clustering, statements on the genetic similarity of infections continue to appear in court cases [69]. On the other hand, clustering can have population-level benefits by enabling public health agencies to rapidly detect elevated rates of transmission in a population, and thereby optimize the allocation of prevention efforts [70]. The expansion of public health applications of clustering [71] has raised concerns among people living with HIV that this use of personal health data might also expose them to a greater risk of criminal prosecution for transmission [72,73].

### Forensic applications of paraphyly methods

**Fig 5:**
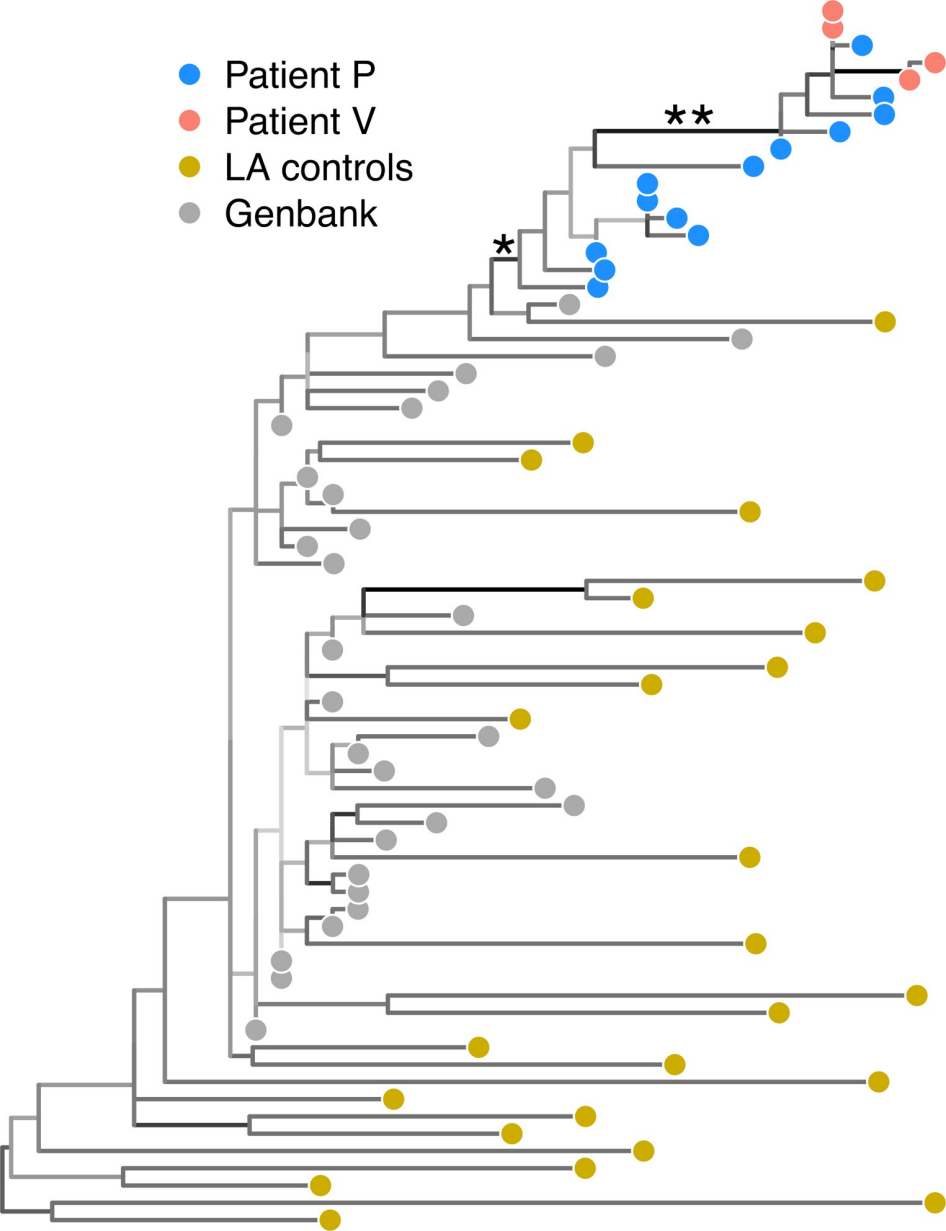
Reproduction of phylogenetic tree from Metzker study. This unrooted tree was reconstructed by maximum likelihood from published HIV-1 RT sequences from Metzker *et al*. [78] and supplemented with additional sequences from Genbank. Tips representing sequences are coloured by source (see legend), and branches are coloured by bootstrap support (darker shades indicate higher support). The branch (labelled ’*’) separating sequences from both patients P and V from the "background" sequences, including the original LA (Louisiana) control sequences from the study, had a support of 95%. The branch (labelled ’**’) cited by the study as evidence that sequences from patient V were nested within a paraphyletic group of sequences from patient P had a support of 100%.

Source attribution methods based on paraphyly have been used in the prosecution of individuals for HIV-1 transmission. One of the earliest examples was published in 2002, where a physician was accused of intentionally injecting blood from one patient (*P*) who was HIV-1 positive into another patient (*V*) who had previous been in a relationship with the physician [74]. This study used maximum likelihood methods to reconstruct a phylogenetic tree relating HIV-1 sequences from both patients. Paraphyly of sequences from *P* implying either direct or indirect transmission to *V* was reported for the phylogeny reconstructed from RT sequences (**Fig 5**). However, a second tree reconstructed from the more diverse HIV-1 envelope (*env*) sequences from the same group was inconclusive on the direction of transmission - only that the *env* sequences from patients *P* and *V* clustered respectively into two monophyletic groups that were jointly distinct from the background.

The use of paraphyly for source attribution was stimulated with the onset of next-generation sequencing, which made it more cost-effective to rapidly sequence large numbers of individual viruses from multiple host individuals. More recent work [41] has also developed a formalized framework for interpreting the distribution of sequences in the phylogeny as being consistent with a direction of transmission. Several studies have since applied this framework to re-analyze or develop forensic evidence for HIV transmission cases in Serbia [75], Taiwan [76], China [77] and Portugal [78]. The growing number of such studies has led to controversy on the ethical and legal implications of this type of phylogenetic analysis for HIV-1 [79].

The accuracy of classifying a group of sequences in a phylogeny into monophyletic or paraphyletic groups is highly contingent on the accuracy of tree reconstruction. As described above (see Paraphyly), our statistical confidence of a specific clade in the tree is quantified by the estimated probability that the same clade would be obtained if the tree reconstruction was repeated on an equivalent data set. This support value is not the probability that the clade appears in the "true" tree because this quantity is conditional on the data at hand - however, it is often misinterpreted this way [80]. If the branch separating a nested monophyletic clade of sequences from host A from the paraphyletic group of sequences from host B has a low support value, then the conventional procedure would be to remove that branch from the tree. This would have the result of collapsing the monophyletic and paraphyletic clades so that the tree is inconclusive about either direction of transmission. However, this procedure has not been consistently used in source attribution investigations. For example, the trees displayed in the 2020 study in Taiwan [76] do not support transmission from the defendent to the plaintiff when branches with low support (<80%) are collapsed. Moreover, the result can vary with the region of the virus genome targeted for sequencing [81].

The use of paraphyly to infer the direction of transmission was recently evaluated on a prospective cohort of HIV serodiscordant couples (where one partner was HIV positive at the start of the study) [82]. Applying the paraphyly method to next-generation sequence data generated from samples obtained from 33 pairs where the HIV negative partner became infected over the course of the study, the authors found that the direction of transmission was incorrectly reconstructed in about 13% to 21% of cases, depending on which sequences were analyzed. However, a follow-up study involving many of the same authors [83] used a more comprehensive sequencing method to cover the full virus genome in depth from all host individuals, lowering the percentage of misclassified cases to 3.1%.

### Forensic applications of phylodynamics

A common feature of both clustering and paraphyly methods is that neither approach explicitly tests the hypothesis that an infection was directly transmitted from a specific source population or individual to the recipient. Phylodynamic methods attempt to overcome the discordance between the pathogen phylogeny and the underlying transmission history by modeling the processes that contribute to this discordance, such as the evolution of pathogen populations within each host. The development of phylodynamic methods for source attribution has been a rapidly expanding area, with a large number of published studies and associated software released since 2014 (see Software). Because these methods have tended to be applied to other infectious diseases including influenza A virus [84], foot-and-mouth disease virus [85] and *Mycobacterium tuberculosis [50]*, they have so far avoided the ethical issues of stigma and criminalization associated with HIV-1. However, applications of phylodynamic source attribution to HIV-1 have begun to appear in the literature. For example, in a study based in Alberta, Canada [86], the investigators used a phylodynamic method (TransPhylo [61]) to reconstruct transmission events among patients receiving treatment at their clinic from HIV-1 sequence data. Although the program TransPhylo attempts, by default, to estimate the proportion of infections that are unsampled, the investigators fixed this proportion to 1%. By so doing, their analysis carried the unrealistic assumption that nearly every person living with HIV-1 in their regional epidemic (comprising at least 1,800 people) was represented in their data set of 139 sequences.

### 2010 cholera outbreak in Haiti

In the aftermath of a magnitude 7.0 earthquake that struck Haiti in 2010, there was a large-scale outbreak of cholera, a gastrointestinal infection caused by the bacterium *Vibrio cholerae*. Nearly 800,000 Haitians became infected and nearly 10,000 died in one of the most significant outbreaks of cholera in modern history. Initial microbial subtyping using pulsed-field gel electrophoresis indicated that the outbreak was most genetically similar to cholera strains sampled in South Asia [87]. In order to more comprehensively map the plausible source of infection, cholera strains from Southern Asia and South America were compared to the strains sampled from the Haitian outbreak. Whole genome sequences taken from cases in Haiti shared more sites in common with the sequences taken from South Asia (*i*.*e*., Nepal and Bangladesh) than those in geographic areas more immediate to Haiti [88]. Direct comparisons were also made between the cholera strains taken from three Nepalese soldiers and three Haitian locals, which were nearly identical in genome sequence, forming a phylogenetic cluster [89]. Based on the evidence gathered by phylogenetic source attribution studies, the role of Nepalese soldiers who were part of the United Nations Stabilization Mission to Haiti (MINUSTAH) in this outbreak was officially recognized by the United Nations in 2016 [90].

### 2019/2020 novel coronavirus outbreak

In December 2019, an outbreak of 27 cases of viral pneumonia was reported in association with a seafood market in Wuhan, China. Known respiratory viruses including influenza A virus, respiratory syncytial virus and SARS coronavirus were soon ruled out by laboratory testing. On January 10, 2020, the genome sequence of the novel coronavirus, most closely related to bat SARS-coronaviruses, was released into the public domain. Despite unprecedented quarantine measures, the virus (eventually named SARS-CoV-2) spread to other countries including the United States, with global prevalence exceeding 556 million confirmed cases as of July 15, 2022 [91].

This outbreak spurred an unprecedented level of epidemiological and genomic data sharing and real-time analysis, which was often communicated by social media prior to peer review. Much of this knowledge translation was mediated through the open-source project Nextstrain [92] that performs phylogenetic analyses on pathogen sequence data as they become available on public and access-restricted databases, and uses the results to update web documents in real time. On March 4, 2020, Nextstrain developers released a phylogeny in which a SARS-CoV-2 genome that was isolated from a German patient occupied an ancestral position relative to a monophyletic clade of sequences sampled from Europe and Mexico. Users of the Twitter social media platform soon commented on the related post from Nextstrain that onward transmission from the German individual seemed to have "led directly to some fraction of the widespread outbreak circulating in Europe today" [93]. These comments were soon followed by criticism from other users that attributing the outbreak in Europe to the German patient as the source individual was drawing conclusions about the directionality of transmission from an incompletely sampled tree [94]. In other words, the tree was reconstructed from a highly incomplete sample of cases from the ongoing outbreak, and the addition of other sequences had a substantial probability of modifying the inferred relationship between the German sequence and the clade in question. Nevertheless, the interpretation attributing the European outbreak to a German source propagated through social media, causing some users to call on Germany to apologize [95].

## Software

There are numerous computational tools for source attribution that have been published, particularly for phylodynamic methods. **Table 1** provides a non-exhaustive listing of some of the software in the public domain. Several of these programs are implemented within the Bayesian software package BEAST [25], including SCOTTI, BadTrIP, and beastlier. This listing does not include clustering methods, which are not designed for the purpose of source attribution, but may be used to develop microbial subtype definitions — clustering methods have previously been reviewed in molecular epidemiology literature [16,96].

**Table 1:** Summary of source attribution software packages in the public domain.

Name	Ref	Method	Input	Software License
outbreaker2	[97]	Phylodynamic, Bayesian	Sampling dates, contacts, genetic sequences	Contributor Code of Conduct
SCOTTI	[58]	Phylodynamic, Bayesian (MCMC, structural coalescent)	Sampling dates, genetic sequences	GNU General Public License v3.0
seqTrack	[84]	Genetic distance clustering (directed graph, maximum parsimony)	Collection dates, genetic distances	GNU General Public License v3.0
sourceR	[98]	Hald model, Bayesian MCMC	Sampling dates, sampling locations, genetic sequences	GNU General Public License v3.0
TransPhylo	[61]	Phylodynamic, Bayesian (MCMC, branching process)	Sampling dates, genetic sequences	GNU General Public License v2.0
PhyloScanner	[99]	Ancestral reconstruction (maximum parsimony)	Short read alignment (BAM format)	GNU General Public License v3.0
QUENTIN	[100]	Phylodynamic, Bayesian (MCMC)	Genetic sequences	GNU General Public License v3.0
genPomp	[101]	Phylodynamic, Bayesian (sequential Monte Carlo)	Sampling dates, genetic sequences	GNU General Public License v3.0
BadTrIP	[102]	Phylodynamic, Bayesian (MCMC)	Sampling dates, genetic sequences, host infectious interval	GNU General Public License v3.0
phybreak	[103]	Phylodynamic, Bayesian (MCMC)	Sampling dates, genetic sequences	GNU General Public License v3.0
TransPairs	[104]	Phylodynamic, maximum-likelihood (optimum branching)	Phylogeny	GNU General Public License v3.0
beastlier	[105]	Phylodynamic, Bayesian	Sequence alignment, non-infectious dates, sample collection dates	None specified

## Supporting information

S1 Text Version history of the text file. (XML)

S2 Text Peer reviews and response to reviews. (XML)
